# Node Attribute-enhanced Community Detection in Complex Networks

**DOI:** 10.1038/s41598-017-02751-8

**Published:** 2017-05-25

**Authors:** Caiyan Jia, Yafang Li, Matthew B. Carson, Xiaoyang Wang, Jian Yu

**Affiliations:** 1 School of Computer and Information Technology & Beijing Key Lab of Traffic Data Analysis and Mining, Beijing Jiaotong University, Beijing, China; 2 Division of Health and Biomedical Informatics, Department of Preventive Medicine, Feinberg School of Medicine, Northwestern University, Chicago, IL USA

## Abstract

Community detection involves grouping the nodes of a network such that nodes in the same community are more densely connected to each other than to the rest of the network. Previous studies have focused mainly on identifying communities in networks using node connectivity. However, each node in a network may be associated with many attributes. Identifying communities in networks combining node attributes has become increasingly popular in recent years. Most existing methods operate on networks with attributes of binary, categorical, or numerical type only. In this study, we introduce *k*NN-enhance, a simple and flexible community detection approach that uses node attribute enhancement. This approach adds the *k* Nearest Neighbor (*k*NN) graph of node attributes to alleviate the sparsity and the noise effect of an original network, thereby strengthening the community structure in the network. We use two testing algorithms, *k*NN-nearest and *k*NN-Kmeans, to partition the newly generated, attribute-enhanced graph. Our analyses of synthetic and real world networks have shown that the proposed algorithms achieve better performance compared to existing state-of-the-art algorithms. Further, the algorithms are able to deal with networks containing different combinations of binary, categorical, or numerical attributes and could be easily extended to the analysis of massive networks.

## Introduction

Complex networks provide a powerful tool for representing real-world complex systems^1^. Social networks, the World Wide Web, protein-protein interaction networks, academic citation and coauthor networks, and hyper-linked blogs are typical examples of such networks, where nodes denote objects and links denote pairs of relations between nodes. In recent years, much effort has been focused on identifying communities, groups of related nodes with dense internal connections and few external connections^2–5^. In addition to node connectivity information, most real-world networks have node-associated attributes. In this case, two types of information are available; graph data to represent the relationship between objects and attribute data to characterize a single object. Thus, nodes can be grouped either by data clustering methods using only their attributes^6^, or by community detection methods using only their link structure^4, 7^. However, clustering objects by attribute similarity ignores relationships between objects, and identifying communities using only links between pairs of nodes isolates node attributes within communities. Therefore, various methods have been developed to uncover communities in networks by combining structural and attribute information such that nodes in a community are not only connected more densely than nodes outside of the community, but also share similar attributes.

Existing methods can be classified roughly into two categories. The first category is composed of probabilistic generative models that formulate joint models of link connections and node attributes, and that use the models to infer the posterior community memberships of nodes in a network^8–17^. The second category contains three types of hybrid methods. The first represents links as a class of node feature and uses node attributes and link connections to perform vertex clustering^18–20^. The second makes use of node attributes to help identify communities in networks^21^. The third uses node attributes and link structure together to optimize a unified objective function^22, 23^.

Probabilistic generative models include CESNA^15^, PCL-DC^9^, PPL-DC^10^, PPSB-DC^11^, cohsMix^12^, BAGC^13^, GBAGC^14^, BNPA^17^, and Metacode^16^. CESNA employs the probabilistic generative process of BIGCLAM^24^ for generating links and the logistic model of attributes together to infer the distribution of community memberships. PCL-DC, PPL-DC, and PPSB-DC project the discriminative content (DC) model of attributes into a generative model of links (like PCL^9^, PPL^10^, and PPSB^11^) via community memberships. cohsMix embeds numerical attributes of nodes into the MixNet model^25^ for generating link classes. BAGC and GBAGC extend the cohsMix model to process categorical attributes and weighted networks. BNPA introduces node attributes and Bayesian priors to Newman’s mixture model^26^ and integrates the Chinese Restaurant Process to infer the number of communities. Metacode represents node attributes as metadata that describe properties of nodes and incorporates the metadata with the degree corrected stochastic block model^27^ to infer correlation between metadata and network structure. These models have good interpretability and provide powerful tools to discover overlapping communities or general structures. However, existing models deal with only one type of attribute (either binary, categorical, or numerical) and are sensitive to initial values.

SA-cluster^18^ and Inc-cluster^19, 20^ are typical examples of vertex clustering methods that use node attributes and link connections. SA-cluster views node attributes as virtual vertices, constructs an attribute-augmented graph, and performs a random walk on the attribute-augmented graph to obtain a unified distance. It then adopts the *K*-medoids algorithm to cluster the nodes based on learned pairwise distance. Inc-cluster was introduced as a slightly faster version of SA-cluster. CODICIL^21^ constructs content edges by selecting the top $\bar{K}$ neighbors of each vertex using their attributes, obtains the combined similarity of a pair of nodes, and then sparsifies the newly constructed graph with content edges^28^. Finally, a fast graph clustering algorithm (Metis^29^ or MLR-MCL^30^) is used to partition the sparsified graph into *K* communities. GLFM^22^ extends MLFM^31^ (the multiplicative latent factor model) to give a unified model of homophily in networks such that an edge is more likely to exist between two nodes with similar attributes than between nodes having different attributes. A minorization-maximization algorithm is then used to optimize the latent eigenmodel of GLMF. PICS^23^ finds cohesive clusters of nodes that have similar connectivity patterns and exhibit high levels of attribute homogeneity by optimizing a unified objective function defined by minimum description length. Compared to probabilistic generative models, these hybrid methods are more efficient. Nonetheless, these methods were designed to process networks with binary or categorical attributes only.

Nearly all of the methods mentioned above follow the assumption that cluster memberships related to node attributes must be consistent with community memberships determined by link structure for a network. However, it is not always true in real world networks. In fact, although nodes in the same community tend to have similar features by the homophily hypothesis^32^, there may exist some nodes in a community that share similar attributes but are not linked due to the sparseness of a real network. Therefore, for each node, we used only a small portion of the nearest neighbors measured by attribute similarity to alleviate the sparsity of a network, while strengthening the community structure. Consequently, in this study, we have proposed a node attribute-enhanced community detection approach, named *k*NN-enhance, using the *k*NN (e.g., *k* ≤ 10) graph of node attributes. We have instantiated *k*NN-enhance into two algorithms, *k*NN-nearest and *k*NN-Kmeans, to test the efficiency and the effectiveness of the approach. In the first stage, we constructed a *k*NN graph enhanced network by adding the *k*NN graph of node attributes to the original network. Then, we selected the number of communities and community centers on the enhanced network using the idea behind the method *K*-rank-D^33^, which is the extended version of the data clustering method proposed by Rodriguez and Laio^34^. In the second stage, we used *k*NN-nearest or *k*NN-Kmeans to cluster nodes into groups, where *k*NN-nearest assigned each remaining node to the cluster of its nearest neighbor with higher centrality and *k*NN-Kmeans clustered nodes iteratively by the *K*-means method. Our experimental results suggest that *k*NN-enhance improves upon existing algorithms through its ability to process networks with binary, categorical, or numerical attributes. Moreover, the approach can handle large-scale attributed networks by combining fast approximate *k*NN-graph algorithms^35–37^ with fast community detection algorithms such as BGLL^38^ and Informap^39^.

## Results

### A Description and Illustration of *k*NN-enhance

**Figure 1:**
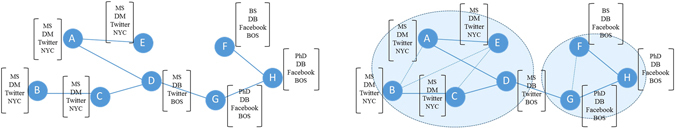
An illustrated example of *k*NN-enhance.

Networks in real applications are often sparse and contain noise in the form of spurious edges. This sparseness and noise blur the community structure of a network. Yet, nodes in the same community are likely to be connected to each other and share similar interests even though some of them are ‘silent’. Therefore, we can obtain a *k*NN graph by using a set of node attributes. The *k*NN-graph is then combined with the original network to compensate for sparsity, thereby strengthening the community structure of the network. Figure 1 is an illustration of *k*NN-enhance. Figure 1a shows an attributed network, where each node has four attributes: degree, research area, affiliation, and location. This original network is sparse and the community structure in it is not clear. If we add a link between nearest neighbors with common node attributes for each pair of nodes (Fig. 1b), the now attribute-enhanced network shows distinctive community structure. Optionally, a community detection algorithm like *K*-rank-D can be used to discover community structure in the newly generated, attribute-enhanced network.

**Figure 2:**
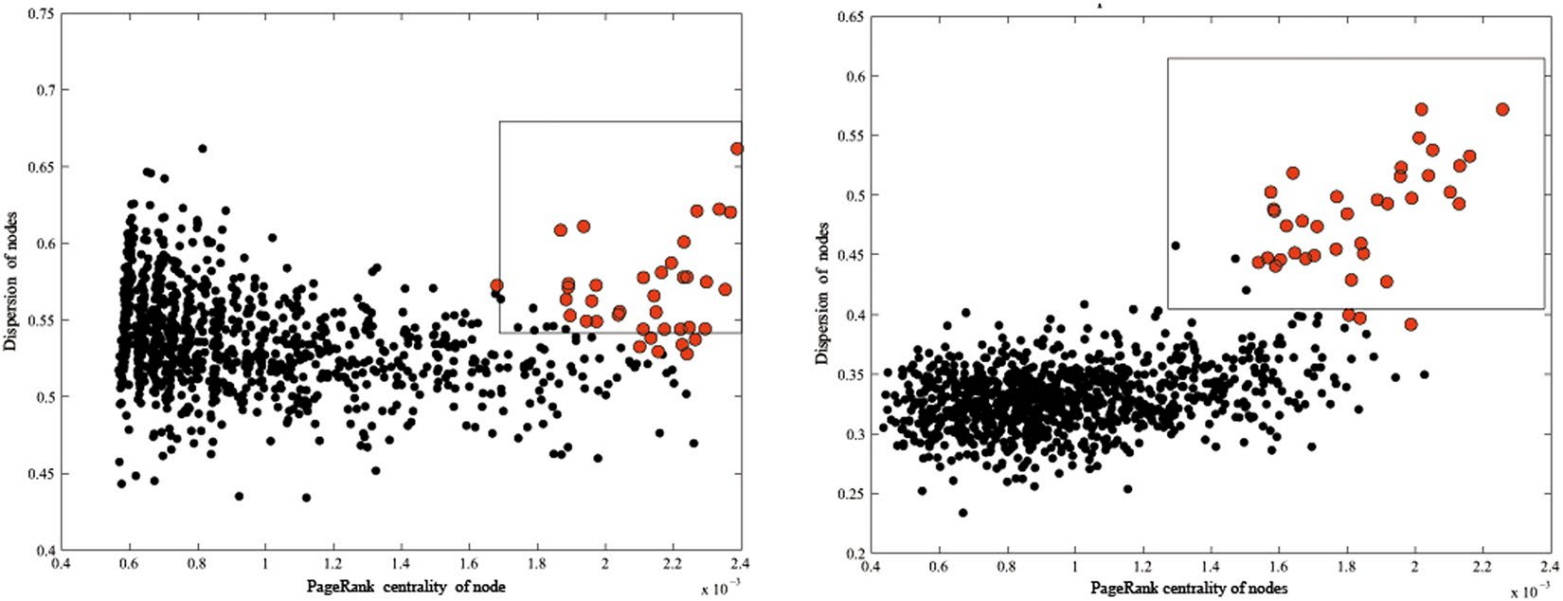
The decision graph of an original LFR network and that of its *k*NN enhanced network.

Figure 2 illustrates the effectiveness of *k*NN-enhance from its partition process. Figure 2a is an example of the decision graph of an original LFR network^40^ with *μ* = 0.9 and *n* = 1000 using *K*-rank-D. The original network contained 38 communities. One hundred binary attributes with the same cluster structure as the original network were attached to each node at a noise ratio of 20%. In the original LFR network, the community structure was unclear and the 38 community centers were not sufficiently separated in the right upper corner of the decision graph. As a result, it was difficult to determine the number of communities and the community centers as well as to detect the community structure in the network. Subsequently, the *k*NN-graph was added to the original network and the decision graph of the *k*NN-graph enhanced network was created with *k* = 10 using *K*-rank-D (Fig. 2b). The community structure became clearer and the 38 community centers were separated in the right upper part of the decision graph. This made the community structure much easier to determine. In addition, the red nodes in Fig. 2b were the top 38 nodes with highest comprehensive value (computed by Equation (4) in the Methods section). The nodes in the square were selected by manually drawing a rectangle in the right upper section of the graph. Using manually selected nodes as initial centers, all nodes are correctly partitioned when compared to the ground truth. Yet, using the top 38 nodes (red nodes) as initial centers, the accuracy (computed by Equation (5) in the Methods section) is only 95%. In some cases it is difficult to select the exact *K* community centers in decision graphs (see Fig. 2a as an example). We automatically selected the top *K* nodes with the highest comprehensive value as the centers in the following experiments.

### Experiment Results

We generated two groups of LFR^40^ benchmark networks with binary and numerical node attributes, respectively. We tested existing state-of-the-art algorithms including probabilistic models (PCL-DC, PPL-DC, PPSB-DC, CESNA, cohsmix, BAGC, and GBAGC) and hybrid methods (SA-Custer, Inc-Cluster, CODICIL, and GLFM) on these synthetic benchmarks. We then evaluated these algorithms on several commonly used real networks, including some with or some without associated ground truth. We compared two instantiations of our *k*NN-enhance approach, *k*NN-nearest and *k*NN-Kmeans, to these existing algorithms. In addition, we compared *k*NN-nearest and *k*NN-Kmeans with *K*-rank-D using only link information, *K*-means using only node attributes, and cluster-dp^34^ using both node attribute and link information on these networks to show whether the proposed approach performed better than existing similar methods and methods using either links or attributes alone.

#### Experimental Results on Synthetic Networks

Largeron *et al*.^41^ have provided a generator to generate networks with community structure and numerical node attributes. However, the generator cannot be used to generate networks with binary attributes. Therefore, we generated our own series of networks based on a commonly used LFR benchmark^40^.

**LFR benchmark networks** are presented by Lancichinetti *et al*.^40^. These mimic real networks by introducing associated characteristics, i.e., the heterogeneity in the distribution of node degree and community size. The LFR benchmark method uses several parameters to generate a network, including *n* (the number of vertices), *μ* (the mixing parameter), 〈*k*〉 (the average degree of vertices), *k*_*max*_ (the maximum degree of vertices), *C*_*min*_ (the minimum community size), *C*_*max*_ (the maximum community size), *γ* and *β* (exponents of the power-law distribution of node degree and community size). The mixing parameter *μ* is designed to control the clearness of community structure in a network. Each node shares a fraction 1 − *μ* of its links with other nodes in its community and a fraction of *μ* with the other nodes in the network. Thus, the smaller *μ* is, the clearer the community structure in an LFR network. When *μ* ≤ 0.6, all algorithms are able to classify nearly all vertices into the correct communities. Therefore, we only added node attributes to LFR networks when *μ* = 0.7, 0.8, or 0.9. Following the example of previous studies^33, 40^, we generated a group of LFR benchmarks with 1000 nodes, $\langle k\rangle =20$, *k*_*max*_ = 50, *C*_*min*_ = 10, *C*_*max*_ = 50, *γ* = 2, and *β* = 1.

We generated two types of node attributes, binary and numerical, for the LFR benchmarks. We did not generate category attributes for simplicity since these can be formulated as binary attributes. We first attached *D*-dimensional binary attributes to each node and gave nodes in the same community the same *d* (*d* < *D*) attributes. In this group of experiments, we set *D* = 100 and *d* = 10 for testing high dimensional attributes. In order to blur the attribute cluster structure, we added 10% to 50% noise by randomly flipping the corresponding portion of binary attributes. With the increase of the noise ratio, the clearness of cluster structure decreased. We then used the Gaussian cluster generator (http://personalpages.manchester.ac.uk/mbs/Julia.Handl) to generate *D* dimensions of numerical attributes following multivariate normal distributions such that the cluster structure of attributes was the same as the community structure of the corresponding network. For a single multivariate cluster, the mean was uniformly distributed in the range [−10, 10], the off-diagonal entries of the covariance matrix were generated as a random number in the range [−1, 1], and the diagonal entries of the covariance matrix were generated as the sum of all off-diagonal entries plus a random number in the range $[\mathrm{0,}\,20\cdot \sqrt{D}]$. We set *D* = 10, 5, 3, and 2 in these groups of experiments. Higher dimensionality led to clearer attribute clusters.

We first compared six probabilistic generative models including PCL-DC, PPL-DC, PPSB-DC, BAGC, GBAGC, and CENSA and seven hybrid methods comprising CODICIL, SA-cluster, Inc-cluster, GLFM, cluster-dp, *k*NN-nearest, and *k*NN-Kmeans on the sample sets with binary attributes, where the noise ratio was in the range $\{\mathrm{10 \% },\mathrm{20 \% ,}\,\cdots ,50 \% \}$ at *μ* = 0.7, 0.8 or 0.9, respectively. Also, we compared all algorithms to *K*-rank-D using only links and *K*-means using only attributes. We reported the average results and standard deviations on 10 sample sets for each setting shown in Tables 1, 2 and 3, where columns indicate the noise ratio of LFR benchmarks, the three numbers in each cell represent the average values and the standard deviations of the three accuracy metrics (ACC, NMI, and PWF defined by Equations (5–7) in the Methods section) of the corresponding algorithm, and the best performing algorithm is marked in bold. The details of parameter settings of these compared algorithms can be found in the Methods section. We did not include the results for PICS and BNPA in the tables because they did not converge to the real number of communities and distorted the meaning of the accuracy metrics (ACC, NMI and PWF).

**Table 1:** Results on LFR networks with binary attributes, *μ* = 0.7.

Algorithms	10%	20%	30%	40%	50%
*K*-rank-D	0.7124 ± 0.0533	0.7124 ± 0.0533	0.7124 ± 0.0533	0.7124 ± 0.0533	0.7124 ± 0.0533
	0.7718 ± 0.0523	0.7718 ± 0.0523	0.7718 ± 0.0523	0.7718 ± 0.0523	0.7718 ± 0.0523
	0.5880 ± 0.0653	0.5880 ± 0.0653	0.5880 ± 0.0653	0.5880 ± 0.0653	0.5880 ± 0.0653
*K*-means	0.9452 ± 0.0166	0.9301 ± 0.0197	0.9355 ± 0.0130	0.8354 ± 0.0271	0.4567 ± 0.0530
	0.9856 ± 0.0043	0.9801 ± 0.0041	0.9667 ± 0.0053	0.8505 ± 0.0192	0.5348 ± 0.0297
	0.9560 ± 0.0149	0.9427 ± 0.0137	0.9343 ± 0.0134	0.7747 ± 0.0345	0.3298 ± 0.0530
PCL-DC	0.9031 ± 0.0324	0.8680 ± 0.0327	0.8621 ± 0.0143	0.8755 ± 0.0376	**0.8647 ± 0.5480**
	0.9752 ± 0.0075	0.9678 ± 0.0070	0.9635 ± 0.0038	**0.9516 ± 0.0120**	**0.9218 ± 0.5831**
	0.9191 ± 0.0306	0.8923 ± 0.0225	0.8836 ± 0.0163	0.8791 ± 0.0358	**0.8413 ± 0.5331**
PPL-DC	0.9496 ± 0.0181	0.9192 ± 0.0180	0.8991 ± 0.0224	0.8562 ± 0.0234	0.8220 ± 0.0263
	0.9848 ± 0.0040	0.9611 ± 0.0104	0.9416 ± 0.0091	0.9013 ± 0.0120	0.8576 ± 0.0142
	0.9585 ± 0.0134	0.9147 ± 0.0238	0.8822 ± 0.0226	0.8200 ± 0.0223	0.7540 ± 0.0283
PPSB-DC	0.7870 ± 0.0407	0.7700 ± 0.0177	0.7139 ± 0.0225	0.6857 ± 0.0253	0.4499 ± 0.2316
	0.9006 ± 0.0199	0.8713 ± 0.0185	0.8246 ± 0.0201	0.7941 ± 0.0246	0.5556 ± 0.2304
	0.7811 ± 0.0443	0.7443 ± 0.0272	0.6687 ± 0.0295	0.6304 ± 0.0417	0.3800 ± 0.2177
BAGC	0.7931 ± 0.0332	0.7368 ± 0.0449	0.6275 ± 0.0440	0.4789 ± 0.0396	0.3430 ± 0.0335
	0.9239 ± 0.0169	0.8810 ± 0.0291	0.7822 ± 0.0385	0.6228 ± 0.0371	0.4705 ± 0.0421
	0.6740 ± 0.0891	0.5196 ± 0.0927	0.3110 ± 0.0594	0.1627 ± 0.0268	0.0977 ± 0.0108 9
GBAGC	0.9353 ± 0.0420	0.9127 ± 0.0499	0.8943 ± 0.0653	0.8659 ± 0.1068	0.7937 ± 0.1663
	0.9812 ± 0.0130	0.9736 ± 0.0164	0.9588 ± 0.0331	0.9364 ± 0.0686	0.8707 ± 0.1308
	0.9345 ± 0.0515	0.9201 ± 0.0484	0.8899 ± 0.0784	0.8320 ± 0.1731	0.7231 ± 0.2427
CESNA	0.9152 ± 0.0232	0.9104 ± 0.0329	0.8988 ± 0.0286	0.8628 ± 0.0265	0.8120 ± 0.0341
	0.9723 ± 0.0081	0.9695 ± 0.0119	0.9595 ± 0.0079	0.9285 ± 0.0129	0.8858 ± 0.0227
	0.9203 ± 0.0248	0.9180 ± 0.0335	0.8955 ± 0.0311	0.8363 ± 0.0312	0.7624 ± 0.0463
CODICIL	0.6883 ± 0.0770	0.6475 ± 0.1326	0.6402 ± 0.1603	0.5420 ± 0.1547	0.3443 ± 0.0837
	0.8445 ± 0.0530	0.7985 ± 0.1015	0.7567 ± 0.1400	0.6339 ± 0.1332	0.4588 ± 0.0683
	0.6193 ± 0.1027	0.5663 ± 0.1596	0.5408 ± 0.1858	0.3988 ± 0.1646	0.1873 ± 0.0632
SA-cluster	0.3789 ± 0.0338	0.3646 ± 0.0344	0.3519 ± 0.0306	0.3340 ± 0.0303	0.3317 ± 0.0312
	0.5072 ± 0.0332	0.4897 ± 0.0334	0.4691 ± 0.0328	0.4457 ± 0.0373	0.4391 ± 0.0293
	0.2595 ± 0.0252	0.2374 ± 0.0245	0.2149 ± 0.0234	0.1880 ± 0.0236	0.1837 ± 0.0211
Inc-cluster	0.3793 ± 0.0339	0.3662 ± 0.0334	0.3544 ± 0.0310	0.3390 ± 0.0278	0.3324 ± 0.0322
	0.5085 ± 0.0335	0.4930 ± 0.0339	0.4735 ± 0.0333	0.4541 ± 0.0364	0.4442 ± 0.0313
	0.2608 ± 0.0256	0.2411 ± 0.0246	0.2196 ± 0.0241	0.1972 ± 0.0223	0.1895 ± 0.0217
GLFM	0.8608 ± 0.0643	0.8283 ± 0.0494	0.7673 ± 0.0494	0.6636 ± 0.0186	0.3997 ± 0.0357
	0.9617 ± 0.0203	0.9454 ± 0.0199	0.9025 ± 0.0227	0.7709 ± 0.0126	0.5310 ± 0.0270
	0.8337 ± 0.1101	0.7541 ± 0.1174	0.6581 ± 0.0981	0.4737 ± 0.0541	0.2002 ± 0.0280
cluster-dp	0.9716 ± 0.0253	0.8265 ± 0.0494	0.6809 ± 0.0433	0.5294 ± 0.0536	0.4464 ± 0.0393
	0.9920 ± 0.0069	0.9423 ± 0.0161	0.8713 ± 0.0202	0.7422 ± 0.0464	0.6757 ± 0.0384
	0.9667 ± 0.0292	0.7945 ± 0.0575	0.6289 ± 0.0570	0.4362 ± 0.0784	0.3385 ± 0.0608
*k*NN-nearest	**0.9977 ± 0.0046**	0.9789 ± 0.0147	**0.9591 ± 0.0160**	0.8818 ± 0.0291	0.7358 ± 0.0480
	**0.9991 ± 0.0018**	0.9930 ± 0.0052	**0.9834 ± 0.0061**	0.9412 ± 0.0113	0.8331 ± 0.0241
	**0.9964 ± 0.0073**	0.9773 ± 0.0164	0.9550 ± 0.0184	0.8583 ± 0.0343	0.6351 ± 0.0551
*k*NN-Kmeans	0.9946 ± 0.0108	**0.9799 ± 0.0137**	0.9486 ± 0.0240	**0.9046 ± 0.0237**	0.8261 ± 0.0454
	0.9988 ± 0.0024	**0.9937 ± 0.0042**	0.9820 ± 0.0080	0.9505 ± 0.0109	0.8756 ± 0.0271
	0.9962 ± 0.0076	**0.9829 ± 0.0107**	**0.9563 ± 0.0206**	**0.9007 ± 0.0262**	0.7764 ± 0.0521

**Table 2:** Results on LFR networks with binary attributes, *μ* = 0.8.

Algorithms	10%	20%	30%	40%	50%
*K*-rank-D	0.2646 ± 0.0318	0.2646 ± 0.0318	0.2646 ± 0.0318	0.2646 ± 0.0318	0.2646 ± 0.0318
	0.3697 ± 0.0381	0.3697 ± 0.0381	0.3697 ± 0.0381	0.3697 ± 0.0381	0.3697 ± 0.0381
	0.1091 ± 0.0154	0.1091 ± 0.0154	0.1091 ± 0.0154	0.1091 ± 0.0154	0.1091 ± 0.0154
*K*-means	0.9378 ± 0.0193	0.9349 ± 0.0142	0.9362 ± 0.0126	0.8182 ± 0.0303	0.4366 ± 0.0534
	0.9836 ± 0.0044	0.9824 ± 0.0044	0.9644 ± 0.0064	0.8434 ± 0.0168	0.5215 ± 0.0261
	0.9494 ± 0.0148	0.9542 ± 0.0105	0.9351 ± 0.0122	0.7546 ± 0.0297	0.3060 ± 0.0540
PCL-DC	0.9074 ± 0.0259	0.9095 ± 0.0203	0.8909 ± 0.0384	**0.8143 ± 0.0384**	**0.6320 ± 0.0398**
	0.9770 ± 0.0068	0.9732 ± 0.0061	0.9481 ± 0.0149	**0.8596 ± 0.0255**	**0.6793 ± 0.0285**
	0.9260 ± 0.0262	0.9204 ± 0.0177	0.8803 ± 0.0386	**0.7418 ± 0.0483**	**0.4636 ± 0.0457**
PPL-DC	0.8089 ± 0.0353	0.6540 ± 0.0433	0.5250 ± 0.0363	0.4249 ± 0.0749	0.3206 ± 0.0158
	0.8719 ± 0.0194	0.7271 ± 0.0293	0.6272 ± 0.0314	0.4964 ± 0.1012	0.4437 ± 0.0221
	0.7370 ± 0.0309	0.4931 ± 0.0469	0.3531 ± 0.0356	0.2955 ± 0.0859	0.1608 ± 0.0147
PPSB-DC	0.5843 ± 0.0550	0.4407 ± 0.0360	0.3005 ± 0.1304	0.1653 ± 0.0879	0.1232 ± 0.0392
	0.7062 ± 0.0392	0.5783 ± 0.0272	0.4239 ± 0.1297	0.2777 ± 0.0971	0.2379 ± 0.0450
	0.4527 ± 0.0575	0.2979 ± 0.0324	0.1777 ± 0.0951	0.0904 ± 0.0535	0.0609 ± 0.0211
BAGC	0.7485 ± 0.0342	0.5887 ± 0.0489	0.3333 ± 0.0352	0.1744 ± 0.0153	0.1094 ± 0.0155
	0.9106 ± 0.0204	0.7500 ± 0.0400	0.4582 ± 0.0362	0.2539 ± 0.0258	0.1450 ± 0.0270
	0.6546 ± 0.0862	0.2984 ± 0.0611	0.0936 ± 0.0126	0.0625 ± 0.0052	0.0573 ± 0.0051
GBAGC	0.8662 ± 0.0589	0.7644 ± 0.0409	0.4509 ± 0.0490	0.2802 ± 0.0315	0.2099 ± 0.0360
	0.9604 ± 0.0191	0.8911 ± 0.0234	0.6021 ± 0.0447	0.4252 ± 0.0350	0.3343 ± 0.0460
	0.8748 ± 0.0612	0.7403 ± 0.0412	0.3071 ± 0.0534	0.1332 ± 0.0232	0.0798 ± 0.0157
CESNA	0.8785 ± 0.0408	0.7830 ± 0.2304	0.7038 ± 0.0438	0.4334 ± 0.0493	0.3035 ± 0.0329
	0.9539 ± 0.0162	0.8558 ± 0.2170	0.7914 ± 0.0373	0.5412 ± 0.0440	0.4245 ± 0.0369
	0.8796 ± 0.0443	0.7683 ± 0.2474	0.6159 ± 0.0590	0.2589 ± 0.0417	0.1397 ± 0.0219
CODICIL	0.6971 ± 0.0924	0.6951 ± 0.1059	0.6303 ± 0.1287	0.4182 ± 0.0869	0.2069 ± 0.0272
	0.8434 ± 0.0739	0.8298 ± 0.0941	0.7337 ± 0.1199	0.5267 ± 0.0749	0.3422 ± 0.0290
	0.6293 ± 0.1143	0.6258 ± 0.1321	0.5165 ± 0.1493	0.2626 ± 0.0780	0.0842 ± 0.0166
SA-cluster	0.3045 ± 0.0235	0.2764 ± 0.0283	0.2575 ± 0.0243	0.2406 ± 0.0247	0.2272 ± 0.0189
	0.4127 ± 0.0335	0.3836 ± 0.0371	0.3590 ± 0.0331	0.3399 ± 0.0357	0.3249 ± 0.0320
	0.1500 ± 0.0146	0.1244 ± 0.0165	0.1074 ± 0.0111	0.0945 ± 0.0122	0.0838 ± 0.0082
Inc-cluster	0.3053 ± 0.0252	0.2782 ± 0.0278	0.2600 ± 0.0247	0.2444 ± 0.0258	0.2300 ± 0.0189
	0.4144 ± 0.0340	0.3875 ± 0.0382	0.3648 ± 0.0344	0.3445 ± 0.0358	0.3298 ± 0.0316
	0.1517 ± 0.0155	0.1273 ± 0.0173	0.1108 ± 0.0124	0.0974 ± 0.0132	0.0864 ± 0.0087
GLFM	0.8322 ± 0.0763	0.7860 ± 0.0900	0.7081 ± 0.0608	0.4477 ± 0.0392	0.2396 ± 0.0175
	0.9478 ± 0.0324	0.9247 ± 0.0390	0.8282 ± 0.0318	0.5733 ± 0.0274	0.3608 ± 0.0193
	0.7625 ± 0.1700	0.6850 ± 0.1834	0.5281 ± 0.1208	0.2130 ± 0.0273	0.0933 ± 0.0091
cluster-dp	0.9693 ± 0.0185	0.7770 ± 0.0491	0.5641 ± 0.0250	0.3430 ± 0.0303	0.2159 ± 0.0171
	0.9908 ± 0.0057	0.9149 ± 0.0191	0.7523 ± 0.0154	0.5187 ± 0.0400	0.3589 ± 0.0312
	0.9656 ± 0.0225	0.7307 ± 0.0689	0.4488 ± 0.0299	0.2134 ± 0.0308	0.1019 ± 0.0119
*k*NN-nearest	**0.9961 ± 0.0060**	0.9742 ± 0.0168	0.9045 ± 0.0259	0.6354 ± 0.0325	0.2877 ± 0.0301
	**0.9987 ± 0.0021**	0.9915 ± 0.0045	0.9601 ± 0.0099	0.7474 ± 0.0222	0.4067 ± 0.0315
	0.9956 ± 0.0072	0.9709 ± 0.0150	0.8929 ± 0.0292	0.5065 ± 0.0442	0.1341 ± 0.0202
*k*NN-Kmeans	0.9951 ± 0.0076	**0.9811 ± 0.0156**	**0.9071 ± 0.0414**	0.7353 ± 0.0406	0.4044 ± 0.0223
	0.9985 ± 0.0023	**0.9946 ± 0.0051**	**0.9640 ± 0.0136**	0.8205 ± 0.0205	0.5037 ± 0.0206
	**0.9960 ± 0.0065**	**0.9851 ± 0.0133**	**0.9113 ± 0.0398**	0.6704 ± 0.0420	0.2469 ± 0.0277

**Table 3:** Results on LFR networks with binary attributes, *μ* = 0.9.

Algorithms	10%	20%	30%	40%	50%
*K*-rank-D	0.1212 ± 0.0060	0.1212 ± 0.0060	0.1212 ± 0.0060	0.1212 ± 0.0060	0.1212 ± 0.0060
	0.2405 ± 0.0257	0.2405 ± 0.0257	0.2405 ± 0.0257	0.2405 ± 0.0257	0.2405 ± 0.0257
	0.0381 ± 0.0017	0.0381 ± 0.0017	0.0381 ± 0.0017	0.0381 ± 0.0017	0.0381 ± 0.0017
*K*-means	0.9396 ± 0.0176	0.9327 ± 0.0232	0.9409 ± 0.0106	0.8293 ± 0.0315	0.4426 ± 0.0549
	0.9831 ± 0.0056	0.9806 ± 0.0077	0.9682 ± 0.0058	0.8534 ± 0.0215	0.5233 ± 0.0265
	0.9491 ± 0.0186	0.9467 ± 0.0214	0.9404 ± 0.0108	0.7718 ± 0.0381	0.3115 ± 0.0504
PCL-DC	0.8708 ± 0.0232	0.6201 ± 0.0511	0.3318 ± 0.0340	0.2191 ± 0.0191	0.1655 ± 0.0072
	0.9185 ± 0.0138	0.7027 ± 0.0459	0.4691 ± 0.0363	0.3598 ± 0.0261	**0.3033 ± 0.0168**
	0.7961 ± 0.0373	0.4407 ± 0.0596	0.1766 ± 0.0217	0.0956 ± 0.0081	0.0632 ± 0.0045
PPL-DC	0.2906 ± 0.0180	0.2158 ± 0.0136	0.1827 ± 0.0068	0.1577 ± 0.0136	0.1440 ± 0.0045
	0.4805 ± 0.0247	0.3880 ± 0.0226	0.3417 ± 0.0207	0.3043 ± 0.0298	0.2829 ± 0.0245
	0.1588 ± 0.0125	0.0995 ± 0.0076	0.0765 ± 0.0039	0.0590 ± 0.0044	0.0508 ± 0.0016
PPSB-DC	0.1513 ± 0.0282	0.1469 ± 0.0289	0.1369 ± 0.0212	0.1175 ± 0.0087	0.1091 ± 0.0098
	0.2802 ± 0.0579	0.2714 ± 0.0529	0.2666 ± 0.0427	0.2291 ± 0.0153	0.2183 ± 0.0228
	0.0711 ± 0.0171	0.0712 ± 0.0150	0.0641 ± 0.0050	0.0582 ± 0.0038	0.0532 ± 0.0057
BAGC	0.6345 ± 0.0417	0.2864 ± 0.0322	0.1266 ± 0.0216	0.0815 ± 0.0099	0.0691 ± 0.0068
	0.8176 ± 0.0207	0.4216 ± 0.0369	0.1774 ± 0.0296	0.0858 ± 0.0303	0.0585 ± 0.0166
	0.4722 ± 0.0741	0.0965 ± 0.0131	0.0591 ± 0.0053	0.0566 ± 0.0041	0.0563 ± 0.0042
GBAGC	0.7391 ± 0.0410	0.3974 ± 0.0511	0.1908 ± 0.0129	0.1409 ± 0.0079	0.1223 ± 0.0130
	0.8804 ± 0.0158	0.5521 ± 0.0476	0.3402 ± 0.0179	0.2683 ± 0.0287	0.2369 ± 0.0380
	0.7240 ± 0.0417	0.2932 ± 0.0561	0.0904 ± 0.0094	0.0582 ± 0.0054	0.0497 ± 0.0043
CESNA	0.7839 ± 0.0349	0.5728 ± 0.0482	0.1783 ± 0.0096	0.1384 ± 0.0051	0.1263 ± 0.0045
	0.8661 ± 0.0247	0.6736 ± 0.0378	0.3121 ± 0.0243	0.2711 ± 0.0226	0.2547 ± 0.0224
	0.7668 ± 0.0396	0.4844 ± 0.0582	0.0683 ± 0.0052	0.0465 ± 0.0029	0.0406 ± 0.0026
CODICIL	0.6681 ± 0.0914	0.6445 ± 0.1196	0.5691 ± 0.1049	0.3121 ± 0.0658	0.1542 ± 0.0175
	0.8123 ± 0.0860	0.7698 ± 0.1164	0.6727 ± 0.0905	0.4373 ± 0.0456	0.2902 ± 0.0148
	0.5917 ± 0.1295	0.5532 ± 0.1571	0.4447 ± 0.1179	0.1753 ± 0.0517	0.0559 ± 0.0110
SA-cluster	0.1818 ± 0.0159	0.1597 ± 0.0146	0.1427 ± 0.0081	0.1338 ± 0.0081	0.1297 ± 0.0073
	0.2932 ± 0.0257	0.2707 ± 0.0292	0.2557 ± 0.0248	0.2464 ± 0.0241	0.2439 ± 0.0218
	0.0624 ± 0.0045	0.0517 ± 0.0035	0.0457 ± 0.0019	0.0431 ± 0.0030	0.0423 ± 0.0029
Inc-cluster	0.1842 ± 0.0163	0.1607 ± 0.0144	0.1447 ± 0.0077	0.1353 ± 0.0085	0.1303 ± 0.0072
	0.2961 ± 0.0265	0.2736 ± 0.0293	0.2583 ± 0.0252	0.2486 ± 0.0246	0.2446 ± 0.0221
	0.0637 ± 0.0049	0.0521 ± 0.0039	0.0459 ± 0.0019	0.0427 ± 0.0028	0.0413 ± 0.0027
GLFM	0.8084 ± 0.0488	0.5866 ± 0.0234	0.3446 ± 0.0417	0.1898 ± 0.0197	0.1398 ± 0.0065
	0.9087 ± 0.0239	0.7160 ± 0.0147	0.4712 ± 0.0358	0.3060 ± 0.0281	0.2405 ± 0.0253
	0.7062 ± 0.1052	0.2989 ± 0.0595	0.1406 ± 0.0273	0.0732 ± 0.0081	0.0546 ± 0.0046
cluster-dp	0.9485 ± 0.0228	0.7380 ± 0.0422	0.4285 ± 0.0455	0.2391 ± 0.0161	0.1438 ± 0.0084
	0.9846 ± 0.0054	0.8854 ± 0.0200	0.6114 ± 0.0256	0.3720 ± 0.0203	0.2533 ± 0.0244
	0.9369 ± 0.0251	0.6882 ± 0.0540	0.3054 ± 0.0463	0.1207 ± 0.0105	0.0588 ± 0.0033
*k*NN-nearest	**0.9861 ± 0.0088**	0.9435 ± 0.0381	0.7617 ± 0.0411	0.2924 ± 0.0302	0.1280 ± 0.0109
	0.9952 ± 0.0025	0.9809 ± 0.0118	0.8650 ± 0.0164	0.4173 ± 0.0265	0.2024 ± 0.0204
	**0.9839 ± 0.0108**	0.9382 ± 0.0413	0.6872 ± 0.0494	0.1466 ± 0.0198	0.0567 ± 0.0040
*k*NN-Kmeans	0.9828 ± 0.0130	**0.9566 ± 0.0263**	**0.8397 ± 0.0392**	**0.4549 ± 0.0452**	**0.1740 ± 0.0127**
	**0.9954 ± 0.0034**	**0.9867 ± 0.0067**	**0.9126 ± 0.0180**	**0.5637 ± 0.0329**	0.2840 ± 0.0212
	0.9836 ± 0.0138	**0.9619 ± 0.0223**	**0.8071 ± 0.0515**	**0.3218 ± 0.0459**	**0.0664 ± 0.0065**

Since only *k*NN-nearest, *k*NN-Kmeans, cohsMix, and cluster-dp can been used to cope with networks having numerical node attributes, we then compared these four algorithms on LFR benchmarks with numerical attributes at different *D* = 10, 5, 3, or 2 when *μ* = 0.7, 0.8, or 0.9. Also, we compared these four algorithms with *K*-means using only numerical attributes (the results of *K*-rank-D using only links can be seen in Tables 1, 2 and 3). The experimental results are shown in Tables 4, 5 and 6, where columns represent the dimension of numerical attribute space (*D* = 10, 5, 3 or 2), the three numbers in each cell represent the average values and the standard deviations of three accuracy metrics (ACC, NMI, and PWF) of the corresponding algorithm over 10 samples, and ‘−’ indicates that cohsMix was trapped in a saddle point. The best algorithm for each column is marked in bold.

**Table 4:** Results on LFR networks with numerical attributes, *μ* = 0.7.

Algorithms	D = 10	D = 5	D = 3	D = 2
*K*-means	0.8159 ± 0.0438	0.7278 ± 0.0259	0.7251 ± 0.0237	0.7267 ± 0.0359
	0.8827 ± 0.0224	0.8442 ± 0.0129	0.8450 ± 0.0125	0.8552 ± 0.0191
	0.7704 ± 0.0502	0.6774 ± 0.0348	0.6858 ± 0.0331	0.6970 ± 0.0472
cohsMix	0.6838 ± 0.0644	0.6397 ± 0.0573	—	—
	0.8057 ± 0.0328	0.7972 ± 0.0384	—	—
	0.6295 ± 0.0689	0.6023 ± 0.0628	—	—
cluster-dp	0.6929 ± 0.0511	0.6499 ± 0.0410	0.5504 ± 0.0613	0.5584 ± 0.0438
	0.8754 ± 0.0238	0.8655 ± 0.0234	0.8244 ± 0.0253	0.8228 ± 0.0220
	0.6363 ± 0.0583	0.6239 ± 0.0577	0.5262 ± 0.0515	0.4826 ± 0.0358
*k*NN-nearest	**0.9275 ± 0.0184**	0.8684 ± 0.0322	0.7507 ± 0.0399	0.5534 ± 0.0291
	0.9642 ± 0.0082	0.9303 ± 0.0146	0.8534 ± 0.0228	0.7329 ± 0.0209
	0.9158 ± 0.0228	0.8473 ± 0.0367	0.6978 ± 0.0499	0.4842 ± 0.0321
*k*NN-Kmeans	0.9266 ± 0.0134	**0.8788 ± 0.0277**	**0.7662 ± 0.0420**	**0.5645 ± 0.0321**
	**0.9661 ± 0.0063**	**0.9325 ± 0.0131**	**0.8641 ± 0.0193**	**0.7412 ± 0.0198**
	**0.9215 ± 0.0202**	**0.8587 ± 0.0320**	**0.7259 ± 0.0466**	**0.4968 ± 0.0383**

**Table 5:** Results on LFR networks with numerical attributes, *μ* = 0.8.

Algorithms	D = 10	D = 5	D = 3	D = 2
*K*-means	0.7974 ± 0.0354	0.7166 ± 0.0332	0.7055 ± 0.0298	0.7339 ± 0.0192
	0.8800 ± 0.0213	0.8380 ± 0.0118	0.8378 ± 0.0119	0.8588 ± 0.0115
	0.7493 ± 0.0435	0.6598 ± 0.0327	0.6555 ± 0.0277	0.6969 ± 0.0270
cohsMix	0.6786 ± 0.0309	0.6425 ± 0.0462	—	—
	0.8063 ± 0.0224	0.8027 ± 0.0194	—	—
	0.6232 ± 0.0418	0.5940 ± 0.0345	—	—
cluster-dp	0.6067 ± 0.0485	0.5751 ± 0.0539	0.4824 ± 0.0391	0.4329 ± 0.0375
	0.8162 ± 0.0271	0.8019 ± 0.0236	0.7740 ± 0.0172	0.7508 ± 0.0198
	0.5409 ± 0.0500	0.5159 ± 0.0557	0.4469 ± 0.0434	0.3843 ± 0.0376
*k*NN-nearest	**0.8764 ± 0.0322**	0.7956 ± 0.0258	0.6539 ± 0.0443	0.5013 ± 0.0339
	0.9322 ± 0.0141	0.8885 ± 0.0105	0.8140 ± 0.0170	0.7093 ± 0.0204
	0.8425 ± 0.0356	0.7498 ± 0.0290	0.5898 ± 0.0478	0.4256 ± 0.0366
*k*NN-Kmeans	0.8729 ± 0.0345	**0.8054 ± 0.0174**	**0.6602 ± 0.0458**	**0.5016 ± 0.0243**
	**0.9360 ± 0.0141**	**0.8934 ± 0.0080**	**0.8220 ± 0.0179**	**0.7188 ± 0.0163**
	**0.8610 ± 0.0329**	**0.7730 ± 0.0256**	**0.6023 ± 0.0524**	**0.4347 ± 0.0328**

**Table 6:** Results on LFR networks with numerical attributes, *μ* = 0.9.

Algorithms	D = 10	D = 5	D = 3	D = 2
*K*-means	0.7917 ± 0.0281	0.7258 ± 0.0486	0.7074 ± 0.0256	0.7367 ± 0.0228
	0.8729 ± 0.0200	0.8464 ± 0.0186	0.8414 ± 0.0146	0.8571 ± 0.0110
	0.7458 ± 0.0382	0.6761 ± 0.0508	0.6667 ± 0.0343	0.6965 ± 0.0204
cohsMix	0.6775 ± 0.0501	0.6626 ± 0.0989	—	—
	0.8016 ± 0.0258	0.7574 ± 0.0925	—	—
	0.6140 ± 0.0525	0.6313 ± 0.0920	—	—
cluster-dp	0.5696 ± 0.0417	0.5260 ± 0.0343	0.4378 ± 0.0392	0.3731 ± 0.0527
	0.7658 ± 0.0210	0.7584 ± 0.0210	0.7326 ± 0.0197	0.6944 ± 0.0402
	0.4928 ± 0.0434	0.4646 ± 0.0386	0.3943 ± 0.0405	0.3198 ± 0.0552
*k*NN-nearest	0.7434 ± 0.0299	0.6651 ± 0.0435	0.5771 ± 0.0321	0.3800 ± 0.0388
	0.8575 ± 0.0167	0.8168 ± 0.0247	0.7628 ± 0.0247	0.6208 ± 0.0316
	0.6655 ± 0.0483	0.5924 ± 0.0514	0.4911 ± 0.0413	0.2853 ± 0.0398
*k*NN-Kmeans	**0.7780 ± 0.0393**	**0.7072 ± 0.0453**	**0.6266 ± 0.0441**	**0.4256 ± 0.0296**
	**0.8822 ± 0.0140**	**0.8432 ± 0.0217**	**0.7940 ± 0.0247**	**0.6670 ± 0.0301**
	**0.7396 ± 0.0373**	**0.6576 ± 0.0540**	**0.5605 ± 0.0461**	**0.3506 ± 0.0343**

We also tested the algorithms on LFR networks with 5000 nodes, $\langle k\rangle =20$, *k*_*max*_ = 50, *C*_*min*_ = 20, *C*_*max*_ = 100, *γ* = 2, and *β* = 1. Because there were too many testing samples and the results were similar to the first group networks with 1000 nodes, we did not report the results of this group of experiments in the manuscript. Instead, to give a glimpse of the time complexity of the compared algorithms, we have reported the time costs of each algorithm on a randomly generated sample containing 40% noise for binary attributes when {*n* = 1000, *C*_*min*_ = 10, *C*_*max*_ = 50}, {*n* = 5000, *C*_*min*_ = 20, *C*_*max*_ = 100}, and {*n* = 10000, *C*_*min*_ = 20, *C*_*max*_ = 200}, respectively, at $\langle k\rangle =20,{k}_{max}=50,\gamma =2,\beta =1$ and *μ* = 0.8 in Table 7. All algorithms were run only once, each number represents the running time of the corresponding algorithm with time unit ‘second’, ‘—’ indicates that the time cost of the corresponding algorithm was beyond 48 hours, and ‘*’ indicates that the algorithm ran out of memory. These experiments were performed on a laptop with an Intel 2.50 GHz processor and 4 GB of main memory running Windows 7.0. CESNA was implemented in C++, CODICIL was implemented in Python and C/C++, cohsMix was implemented in R, and the remaining algorithms were implemented in MATLAB.

**Table 7:** Time cost of the compared algorithms (in seconds).

Probabilistic models	*n*			Hybrid methods	*n*		
	1000	5000	10000		1000	5000	10000
PCL-DC	716.21	40,718.24	126,607.01	SA-cluster	3.05	67.47	1127.13
PPL-DC	2131.74	103,140.25	—	Inc-cluster	1.56	33.57	645.49
PPSB-DC	8360.91	*	*	CODICIL	7.65	179.74	726.96
CESNA	837.05	5523.21	10,798.43	GLFM	11.68	159.89	588.19
BAGC	0.63	15.72	117.95	cluster-dp	2.07	21.76	351.16
GBAGC	0.51	5.40	21.98	*k*NN-nearest	2.07	22.40	365.42
				*k*NN-Kmeans	2.13	33.23	401.29

From the data in Tables 1–7, we have concluded that adding node attributes promotes the performance of community detection in most cases. Taking the results of *k*NN-Kmeans as an example, most of the results were better than those of the basic K-rank-D algorithm on links and *K*-means on attributes. As Tables 1–3 show, in most cases, *k*NN-nearest and *k*NN-Kmeans performed best among the 13 tested algorithms including probabilistic generative models and hybrid methods, and these outperformed the other hybrid methods in all cases. Although *k*NN-nearest performed slightly worse than *k*NN-Kmeans, it was more efficient (see Table 7) since each node received its community label from the nearest node with higher centrality. According to our experiments, *k*NN-Kmeans converged quickly since community centers were carefully selected. In some cases, the probabilistic generative model PCL-DC displayed the best performance but ran too slowly to be used for processing large networks in real applications (see Table 7). CESNA and CODICIL also showed good performance on this group of experiments. Among the probabilistic methods, CENSA was the fastest algorithm. However, it was much slower than the majority of the hybrid heuristic methods. CODICIL ran quickly due to the fast graph partition program Metis, which was used to cut the networks into communities. GBAGC performed well because it used Metis on links to get the initial partition. Moreover, as Tables 4–6 show, both the *k*NN-nearest and the *k*NN-Kmeans algorithms allowed us to discover communities effectively in LFR networks with numerical attributes. In summary, this empirical study on LFR benchmarks proves the flexibility, effectiveness, and efficiency of the *k*NN-enhance approach.

#### Experimental Results on Real Networks

In addition to our experiments using synthetic networks, we tested the algorithms on two groups of real networks. The nodes in the first group were associated with binary/categorical attributes, while those in the second group possessed numerical attributes. The first group of data sets included Cora^42^, Citeseer^42^, and DBLP10K^18^. Sinanet (https://github.com/smileyan448/Sinanet) and PubMed (http://linqs.umiacs.umd.edu/projects//projects/lbc/) belonged to the second group. Detailed information on these data sets is described below.

**The Cora data set** consisted of machine learning papers. These papers were classified as belonging to one of the following seven classes: CBR (case based reasoning), GA (genetic algorithms), NN (neural networks), PM (probabilistic methods), RL (reinforcement learning), or RLT (rule learning theory). The papers were selected in such a way that in the final corpus every paper cited or was cited by at least one other paper. Assuming each node represented a paper, there were 2,708 nodes and 5,429 citations. After stemming and removing stop-words and words with document frequency less than 10, the corpus remained a vocabulary of size 1,433 unique words. Each paper was described by a 1433-dimension 0/1 vector indicating the absence/presence of the corresponding words from the dictionary of these unique words.

**The Citeseer data set** was also a citation network in the field of machine learning. These papers were classified into one of the following six classes: Agents, AI (artificial intelligence), DB (database), IR (information retrieval), ML (machine learning), and HCI (human-computer interaction). The papers were selected in the same way as the Cora dataset. There were 3,312 papers in the corpus and 4,732 citations between papers. A paper was described by a 0/1 word vector indicating the absence/presence of the corresponding words from the dictionary of the 3,703 unique words.

**The DBLP data set** was a co-author network extracted from DBLP Bibliography data. This network contained 10,000 authors and their coauthor relationships. These authors were distributed across four research fields including databases, data mining, information retrieval, and artificial intelligence. Each author was associated with two relevant attributes; prolific and primary topic. The attribute prolific had three possible values: authors with ≥20 publications were labeled as highly prolific, authors with ≥10 and <20 papers were labeled as prolific, and authors with <10 papers were labeled as low prolific. The attribute primary topic had 99 values. Each author was assigned a primary topic out of 99 extracted by a topic model from a collection of paper titles of the authors. For this data set, we did not know the exact number of communities or to which community a node belonged.

**The Sinanet data set** was a microblog user relationship network that we extracted from the sina-microblog website (http://www.weibo.com). We first selected 100 VIP sina-microblog users distributed across 10 major forums including finance and economics, literature and arts, fashion and vogue, current events and politics, sports, science and technology, entertainment, parenting and education, public welfare, and normal life. Starting from these 100 VIP sina-microblog users, we extracted the followees of these users and their published micro-blogs. Using a depth-first search strategy, we extracted three-layers of user relationships and obtained 8,452 users, 147,653 user relationships, and 5.5 million micro-blogs in total. We merged all microblogs that a user published to characterize that user’s interests^43^. After removing silent users (those who post less than 5000 words), we were left with 3,490 users and 30,282 relationships. If we used words’ frequency of the merged blogs of a user to describe the user’s interest, the dimension of the feature space would have been too high to be successfully processed. We used users’ topic distribution in the 10 forums, which was obtained by the LDA topic model (http://gibbslda.sourceforge.net/), to describe users’ interests. Thus, besides the followee relationships between pairs of users, we have 10 dimensional numerical attributes to describe the interests of each user. This data set is available at https://github.com/smileyan448/Sinanet.

**The Diabetes data set** consisted of 19,717 scientific publications from the PubMed database pertaining to diabetes classified into one of three classes: Diabetes Mellitus Experimental, Diabetes Mellitus Type 1, and Diabetes Mellitus Type 2. These publications formed a citation network with 44,338 edges representing the citation relationships of pairs of publications. Further, each publication in the dataset was described by a TF/IDF weighted word vector from a dictionary that consisted of 500 unique words.

**Figure 3:**
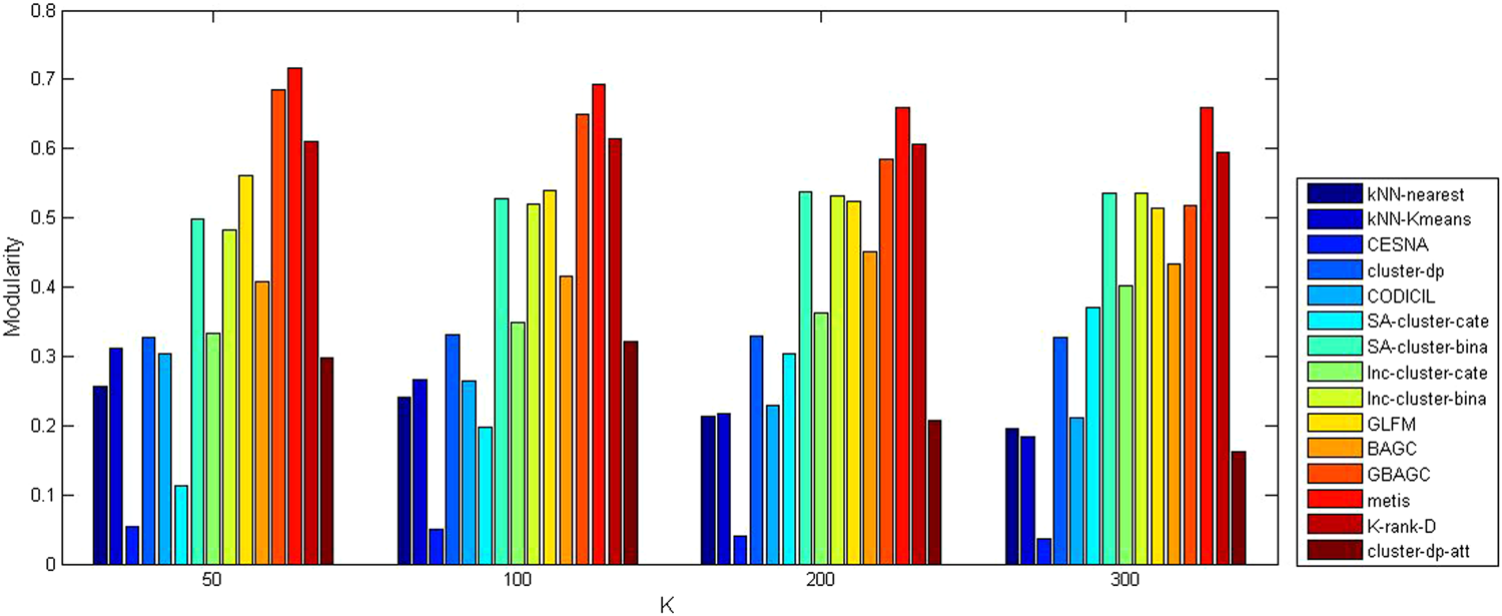
*Modularity* of the compared algorithms on DBLP10k.

**Figure 4:**
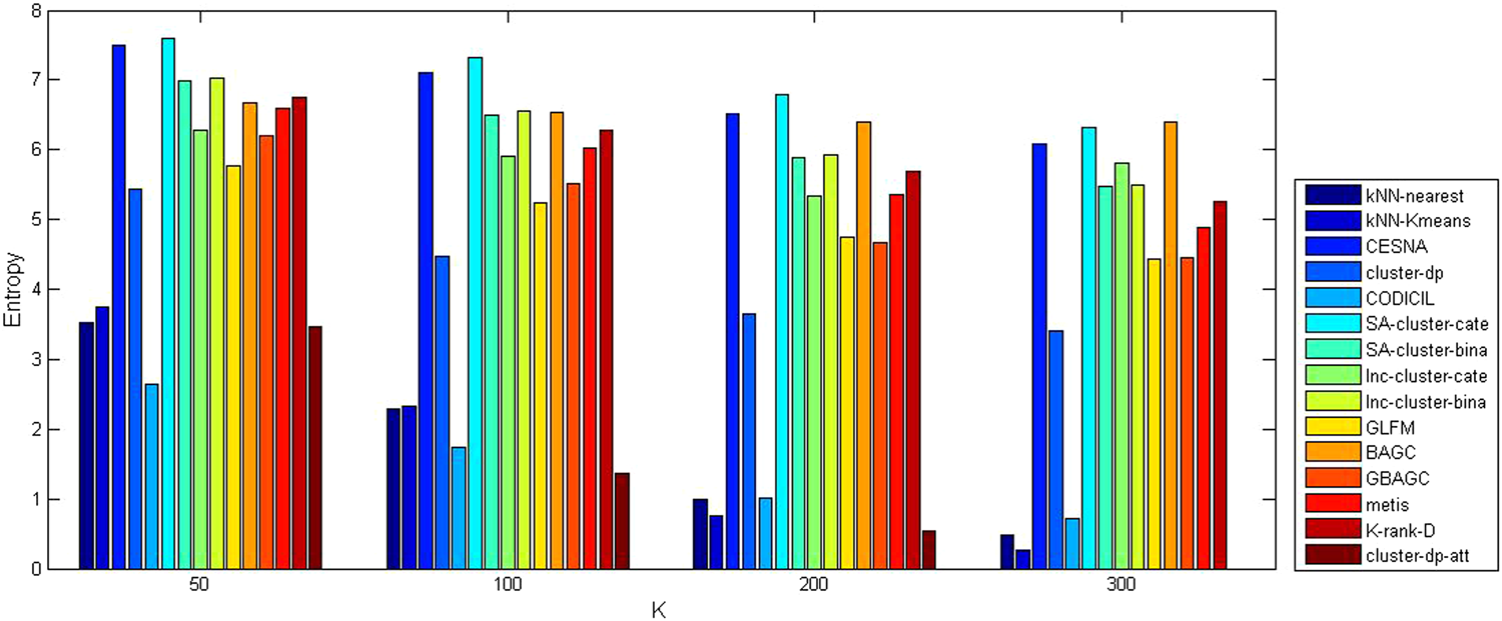
*Entropy* of the compared algorithms on DBLP10k.

The experimental results of the methods on Cora and Citeseer are shown in Table 8, where columns represent the data sets used in the evaluation, the cells of each row represent the values of ACC, NMI, and PWF for the corresponding algorithm, and the algorithm with the best performance is marked in bold in each of the two groups (probabilistic methods and hybrid methods). Because the DBLP network had two categorical attributes, we tested SA-cluster on the two category attributes with 3 values and 99 values, respectively. We named the result SA-cluster-cate. We also tested SA-cluster on DBLP when we viewed these 102 values as binary and named the result SA-cluster-bina (the same was done for Inc-cluster). Since no ground truth was available on DBLP, we reported *Modularity* and *Entropy* (defined by Equations (8–9) in the Methods section) of all algorithms in Figs 3 and 4 at different *K* (*K* is the number of communities). In Figs 3 and 4, we do not show the results of PCL-DC, PPL-DC, and PPSB-DC since either the time or space complexity of these algorithms was too high to handle large networks like DBLP10k. The results of the methods for processing Sinanet and PubMed networks with numerical attributes are shown in Table 9, where ‘*’ indicates that the algorithm ran out of memory on the corresponding data set. For the probabilistic methods PCL-DC, PPL-DC, PPSB-DC on Cora and Citeseer, and cohsMix on Sinanet, we ran the algorithms 10 times and reported the result with the largest likelihood. For *K*-means on attributes alone, we reported the best results of these networks over 10 runs. The details of the parameter settings for the compared algorithms in this group of experiments can be found in the Methods section.

**Table 8:** Performance of compared algorithms on Cora and Citeseer.

Algorithms	Cora			Citeseer		
	ACC	NMI	PWF	ACC	NMI	PWF
*K*-means	0.4136	0.2334	0.3068	0.5344	0.2712	0.3727
*K*-rank-D	0.4668	0.3266	0.3442	0.3469	0.1757	0.2983
PCL-DC	0.5539	0.4005	0.4330	0.4043	0.1703	0.2992
PPL-DC	0.6270	0.4781	0.5233	**0.6380**	**0.4420**	**0.5278**
PPSB-DC	**0.7160**	**0.5264**	**0.5878**	0.5927	0.3402	0.4522
BGAC	0.2895	0.1524	0.2947	0.2316	0.0382	0.3002
GBGAC	0.5654	0.4477	0.4575	0.4303	0.2029	0.3205
CESNA	0.4856	0.2689	0.3794	0.2132	0.0225	0.3023
CODICIL	0.5639	0.3678	0.4044	0.5432	0.2860	0.3936
SA-cluster	0.2637	0.1190	0.2825	0.2325	0.0466	0.2984
Inc-cluster	0.2637	0.1190	0.2825	0.2325	0.0466	0.2984
GLFM	0.6104	**0.5029**	0.4848	**0.6621**	**0.3973**	**0.5094**
cluster-dp	0.4974	0.2800	0.4204	0.3581	0.1440	0.3236
*k*NN-nearest	0.4878	0.3469	0.4403	0.4955	0.2518	0.3819
*k*NN-Kmeans	**0.6662**	0.4569	**0.5014**	0.6301	0.3703	0.4749

**Table 9:** Performance of compared algorithms on Sinanet and PubMed Diabetes.

Algorithms	Sinanet			PubMed Diabetes		
	ACC	NMI	PWF	ACC	NMI	PWF
*K*-means	0.7989	0.6664	0.7080	0.5971	0.3198	0.5855
*K*-rank-D	0.3361	0.1900	0.2994	0.4088	0.0701	0.5012
cohsMix	**0.6957**	**0.5789**	**0.6512**	*	*	*
cluster-dp	0.4166	0.3180	0.3518	0.4007	0.0041	0.5117
*k*NN-nearest	0.3464	0.3172	0.3079	0.5249	0.0745	0.4585
*k*NN-Kmeans	0.6338	0.5356	0.5160	**0.6232**	**0.2162**	**0.5127**

We drew the following conclusions using the information in Tables 8 and 9 and Figs 3 and 4: (1) According to Table 8, probabilistic methods PCL-DC, PPL-DC, and PPSB-DC showed the best performance on Cora and Citeseer data sets. However, the time cost of these methods was too high and they would not be appropriate for real applications. In contrast, the *k*NN-enhance approach achieved high accuracy in comparison to other hybrid methods and was much faster than probabilistic methods PCL-DC, PPL-DC, and PPSB-DC (see Table 7). (2) By Figs 3 and 4, the *Entropy* of *k*NN-Kmeans on DBLP was the lowest, especially when the number of communities *K* was larger than 200. The *Modularity* of *k*NN-enhance indicates that the partitioned network maintained community structure. Therefore, *k*NN-enhance was able to identify a clear community structure (large *Modularity*) with a high level of attribute homogeneity (low *Entropy*) in the network. (3) The *k*NN-enhance approach was capable of processing networks with numerical attributes (see Table 9). Even through the accuracy of cohsMix was higher than *k*NN-nearest and *k*NN-Kmeans on Sinanet, cohsMix ran much slower than *k*NN-enhance and the results from this algorithm were selected over 10 runs on Sinanet data. Moreover, cohsMix was not capable of dealing with a large network such as the one from the Diabetes data set due to its high memory usage when storing the similarity matrix and all hidden variables.

## Discussion

We have proposed a simple and flexible node attribute enhanced community detection approach, *k*NN-enhance. This method was designed to construct the *k* nearest neighbor graph of node attributes first, then merge the *k*NN graph with the original network. With this approach we were able to alleviate the sparsity of the original network, reduce noise effects, and strengthen the community structure of the original network. Because of this, a clear community structure could be partitioned within the *k*NN graph enhanced network by a community detection algorithm like K-rank-D. Our two implementations, *k*NN-nearest and *k*NN-Kmeans, have shown that the proposed algorithms achieved better performance against the existing state-of-the-art algorithms. Furthermore, the algorithms were able to deal with a network containing binary, categorical, or numerical attributes and could be easily extended to process large-scale networks.

In the future we intend to test this approach on large scale networks with millions of edges by combining fast approximate *k*NN graph construction algorithms (such as NN-Descent^36^ with *O*(*n* ^1.14^) empirical cost) with fast community detection algorithms such as BGLL^38^ and Informap^39^. Moreover, besides strengthening the community structure of a network using node attributes, we plan to design a more effective method by removing some easily detected weak-linked edges from the network. In this study we were concerned with detecting community structures containing nodes with more links to each other than to nodes outside their communities. However, it has been observed that trees and tree-like networks have high modularity^44, 45^, the classical objective function to discover communities and to measure their strength^46^, and that many real world networks have tree-like structures^47–49^. Existing methods use connections only to decompose a network into tree-like components. It is a challenging task to combine node attributes with topology to cluster nodes in a tree-like network into groups, and we will investigate whether our *k*NN-enhance approach is capable of partitioning attributed tree-like networks.

## Methods

### Community Detection in Attributed Networks

Suppose that *G* = (*V*, *E*, *X*) is a network with node attributes, where *V* is a set of nodes ($\Vert V\Vert =n$), *E* is an edge set that indicates relationships between pairs of nodes ($\Vert E\Vert =m$) and is usually represented by an adjacency matrix *A* = [*A*_*ij*_] (*A*_*ij*_ = 1 if there is an edge between nodes *i* and *j*, *A*_*ij*_ = 0 otherwise), *X* = {*x*_1_, *x*_2_, …, *x*_*n*_} $({x}_{i}=({x}_{i1},{x}_{i2},\cdots ,{x}_{iD}),i=1,2,\cdots ,n)$ is a set of vectors, each of which denotes the values of *D* attributes associated with a node *i*. We call this an ‘attributed network’ or ‘attributed graph’. Community detection in an attributed network involves partitioning nodes into clusters such that nodes in the same cluster are not only densely connect to each other but also exhibit a high level of attribute homogeneity.

### An Active Method for Community Detection in Networks

cluster-dp is a recently-developed clustering algorithm similar to the *K*-means method^34^. The algorithm assumes that cluster centers are surrounded by neighbors with lower local density and that they are a relatively large distance from any data points with a higher local density. Therefore, for each data point *i*, two quantities, the local density *ρ*_*i*_ and the distance from points of higher density *δ*_*i*_, are defined as follows to quantify the likelihood of a data point being a cluster center:

1 $${\rho }_{i}=\sum _{j}\chi ({d}_{ij}-{d}_{c}),{\delta }_{i}=mi{n}_{j:{\rho }_{j} > {\rho }_{i}}({d}_{ij})$$

where *d*_*ij*_ is the distance of data points *i* and *j*, *χ*(*x*) = 1 if *x* < 0 and *χ*(*x*) = 0 otherwise, *d*_*c*_ represents the cutoff distance, and *δ*_*i*_ = *max*_*j*_(*d*_*ij*_) for the point with the highest density.

If we scatter all data points on a decision graph drawn by their values of *ρ*_*i*_ and *δ*_*i*_ for all $i\in \{1,2,\cdots ,n\}$, the cluster centers tend to occupy the right upper part of the graph. After cluster centers with both relatively large *ρ*_*i*_ and *δi* are manually selected on the decision graph, each remaining point is assigned to the same cluster as its nearest neighbor of higher density. This allows cluster-dp to uncover the cluster structure of data points by actively knowing the number of clusters and cluster centers.

However, the following issues exist: (1) When the cluster structure is not clear (i.e., there is not a distinguished boundary between cluster centers and other data points on the decision graph), it is difficult to obtain the correct number of clusters and cluster centers. This leads to poor partitioning. (2) The parameter *d*_*c*_ must be tuned in many cases, and it is usually difficult to know which parameter value is best. (3) The input for cluster-dp is a distance matrix. The quality of the matrix has a strong effect on the clustering result. When the algorithm is used to discover community structure in a network, the topological structure implied in the network is not fully utilized.

In a network structure, we suppose that community centers are: (1) influential and surrounded by less influential nodes, and (2) located far from each other in the network. Therefore, we have proposed *K*-rank-D^33^ and use two quantities, ${v}_{i}\in v=\{{v}_{1},{v}_{2},\cdots ,{v}_{n}\}$ and ${\bar{\delta }}_{i}$, to describe the centrality and the dispersion of each node *i*, respectively. The centrality vector *v* can be calculated efficiently using PageRank^50^ centrality as follows:

2 $${v}^{t+1}=((1-\beta )P+e\frac{\beta }{n}){v}^{t},\,\,{P}_{ij}=\frac{{A}_{ij}}{{\sum }_{j}{A}_{ij}},\,i,j\in \{1,2,\cdots ,n\}$$

where *β* is the re-start probability (fixed at 0.15), *e* is the unit matrix, *v*_0_ is a *n*-dimensional unit vector, and *v* ^*t*^ is normalized to 1 in each iteration. The dispersion of a node *i* to other nodes with higher centrality is defined by ${\bar{\delta }}_{i}=mi{n}_{j:{v}_{j} > {v}_{i}}({d}_{ij})$ and ${\bar{\delta }}_{k}=max({\bar{\delta }}_{i})$, *i* ≠ *k*, for the node *k* with highest centrality. *d*_*ij*_ is the structural distance between nodes *i* and *j*. It can be computed using Euclidean distance measurement ${\Vert \cdot \Vert }_{2}$ after *τ*-step signal propagation^51^ by following equations:

3 $$S={(A+I)}^{\tau },\,\,{\bar{S}}_{ij}={S}_{ij}/\sqrt{\sum _{j}{S}_{ij}^{2}},\,\,{d}_{ij}={\Vert {\bar{S}}_{i}-{\bar{S}}_{j}\Vert }_{2}.$$

where *τ* = 3 in implementation and ${\bar{S}}_{i}$ is the *i*-th row of $\bar{S}$. In the case that the community structure of a network is fuzzy, we define the comprehensive value for each node *i* as follows:

4 $$CV(i)={v}_{i}\cdot {\bar{\delta }}_{i}/({ma}{{x}}_{j=1}^{n}({v}_{j})\cdot {ma}{{x}}_{j=1}^{n}({\bar{\delta }}_{j})).$$

The top *K* nodes with the highest comprehensive value can then be automatically selected as the initial centers of *K*-rank-D.

### *k*NN-enhance: a Node Attribute-enhanced Community Detection Approach

Given an attributed network *G* = (*V*, *E*, *X*), we first construct the *k*NN graph of node attributes. The *k*NN graph for a set of nodes *V* is a directed graph with vertex set *V* and an edge from each *v* ∈ *V* to its *k* most similar objects in *V* under a given similarity measure on attributes. ∀*x*_*i*_, *x*_*j*_ ∈ *X*, the cosine similarity ${x}_{i}\cdot {x}_{j}^{T}$ is used to compute the similarity of a pair of nodes with binary attributes, and $1-norm({\Vert {x}_{i}-{x}_{j}\Vert }_{2})$ is used to compute the similarity of a pair of nodes with numerical attributes, where $norm({\Vert {x}_{i}-{x}_{j}\Vert }_{2})$ is the normalization of the Euclidean distance of *x*_*i*_ and *x*_*j*_. We then add the *k*NN-graph of attributes to the original network. For an edge of the *k*NN graph, if it is a new edge in the original network, we add this edge to the original network; otherwise, we keep the edge in the original network unchanged.

After the *k*NN-enhanced network is established, we use the *K*-rank-D method introduced above to perform node clustering. In addition to *K*-rank-D, we employ two node assignment strategies after selecting *K* community centers from the decision graph. *k*NN-nearest uses the cluter-dp strategy^34^, which involves assigning each remaining node to the same cluster as its nearest neighbor of higher PageRank centrality computed by Equation (2). *k*NN-Kmeans uses the strategy of the *K*-means method, where the input is the data matrix $\bar{S}=[{\bar{S}}_{ij}]$ ^51^. It iteratively updates its community centers. It should be pointed out that *k*NN-nearest and *k*NN-Kmeans are just two implementations of *k*NN-enhance approach. Fast approximate *k*NN graph construction methods^35–37^ and highly-efficient community detection algorithms^38, 39^ can be combined to process large scale networks.

### Metrics for Evaluating Algorithm Quality

In this study, we use two groups of metrics to evaluate the performance of each algorithm. The first group includes ACC (Accuracy), NMI (Normalized Mutual Information), and PWF (Pairwise F-Measure)^9, 11^. These are commonly used to evaluate an algorithm running on a data set with ground truth. Larger values indicate better algorithm performance. The other group consists of *Modularity* ^38, 46^ and *Entropy* ^13, 18^. *Modularity* is used to measure the quality of communities in a network, and a larger *Modularity* value indicates better partition quality. *Entropy* is used to measure the degree of attribute consistency in a community, and a lower *Entropy* value indicates a greater consistency. These metrics are often used when an algorithm is run on a network without ground truth. Formal definitions are provider below:

**ACC**. Given node *i*, *l*_*pi*_ is the node label assigned by an algorithm and *l*_*ti*_ is its true label. The accuracy is defined by

5 $$ACC=\sum _{i=1}^{n}\delta ({l}_{ti},{p}_{map}({l}_{pi}))/n$$

where *δ*(·) is a Kronecker function, *P*_*map*_(*l*_*pi*_) is a permutation mapping function that maps the label *l*_*pi*_ to its corresponding label *l*_*ti*_ in the ground truth, and *n* is the total number of nodes in a network.

#### NMI

Suppose $C=\{{C}_{1},{C}_{2},\cdots ,{C}_{K}\}$ is a set of *K* communities contained in a network and $C^{\prime} =\{{C}_{1}^{^{\prime} },{C}_{2}^{^{\prime} },\cdots ,{C}_{K}^{^{\prime} }\}$ is a set of *K* communities obtained by a specific algorithm. NMI is defined by

6 $$NMI(C,C^{\prime} )=\frac{-2{\sum }_{i=1}^{K}{\sum }_{j=1}^{K}{n}_{ij}\,\mathrm{log}\frac{n\cdot {n}_{ij}}{{n}_{i}^{C}\cdot {n}_{j}^{{C}^{^{\prime} }}}}{{\sum }_{i=1}^{K}{n}_{i}^{C}\,\mathrm{log}\frac{{n}_{i}^{C}}{n}+{\sum }_{j=1}^{K}{n}_{j}^{{C}^{^{\prime} }}\,\mathrm{log}\frac{{n}_{j}^{{C}^{^{\prime} }}}{n}}$$

where *n*_*ij*_ is the number of nodes in the ground truth community *C*_*i*_ that are assigned to the computed community *C*′_*j*_, ${n}_{i}^{C}$ is the number of nodes in the ground truth community *C*_*i*_, and ${n}_{j}^{{C}^{^{\prime} }}$ is the number of nodes in the computed community *C*′_*j*_.

#### PWF

Let *T* denote the set of nodes in the ground truth communities and *W* denote the set of nodes assigned by a given algorithm in the corresponding communities. PWF is defined as follows:

7 $$PWF=\frac{2\times precision\times recall}{precision+recall}$$

where $precision=\Vert W\cap T\Vert /\Vert W\Vert$, $recall=\Vert W\cap T\Vert /\Vert T\Vert$, and $\Vert \cdot \Vert$ denotes the cardinality of a set.

#### Modularity

Given a network with *n* nodes and *m* edges, *Modularity* can be calculated as follows:

8 $$Modularity=\frac{1}{2m}\sum _{i,j}({A}_{ij}-\frac{{k}_{i}\cdot {k}_{j}}{2m})\delta ({c}_{i},{c}_{j})$$

where *A* = [*A*_*ij*_] is the adjacency matrix of the network, *k*_*i*_ is the degree of node *i*, *δ*(·,·) is the Kronecker function, and *c*_*i*_ is the community to which the node *i* belongs.

#### Entropy

Given a network with *n* nodes, we suppose that each node is associated with *D* attributes $({a}_{1},{a}_{2},\cdots ,{a}_{D})$ and that the nodes can be partitioned into *K* communities. Let *n*_*c*_ be the number of nodes in the *c*-th community and *p*_*ic*_ be the fraction of nodes in the *c*-th community taking attribute *a*_*i*_. The total *Entropy* of attributes in communities can then be defined in the following way:

9 $$Entropy=\sum _{c=1}^{K}\frac{{n}_{c}}{n}\sum _{i=1}^{D}{p}_{ic}\,\mathrm{log}({p}_{ic})$$

*Entropy* measures the homogeneity of communities and their shared attributes.

### Parameter Settings on Synthetic and Real-world Networks

As mentioned above, the PCL-DC, PPL-DC and PPSB-DC methods are sensitive to initial values. For the experiments on these algorithms using synthetic attributed networks, we ran the algorithms 10 times on each sample set, selected the best result determined by maximum likelihood, and then reported the average results and standard deviations on 10 samples. We set the max iteration number and the convergence threshold of PCL-DC, PPL-DC, and PPSB-DB to 2000 and 10^−8^, respectively. We set the regularization coefficient *λ* = 1 for PCL-DC and CESNA and *λ* = 0.1 for PPL-DC and PPSB-DC since they perform the best when *λ* is set accordingly. Similarly, for *K*-means on attributes, we ran it 10 times on each sample set (since it is sensitive to its initial values), selected the best result with the highest accuracy, and then reported the average results and standard deviations on 10 samples. For BAGC and GBAGC, the max iteration number was set at 10. For CODICIL, we set $\bar{K}=30,50$ and 70 and selected the one with the highest accuracy. We used cosine similarity ${x}_{i}\cdot {x}_{j}^{T}$ and signal similarity $1-norm({\Vert {\bar{S}}_{i}-{\bar{S}}_{j}\Vert }_{2})$ to compute the similarity of node attributes and that of link structure for cluster-dp, respectively. We set the weight *α* of attribute similarity and link similarity to 0.5 for cluster-dp and CODICIL since it was difficult to tune the weight adaptively for each sample. We set $\bar{D}=50$ for GLFM. For *k*NN-nearest and *k*NN-Kmeans, we set *k* = 10 because we wanted to strengthen only the community structure of the original network so that small *k* is sufficient. We used default values for algorithm parameters not mentioned above. Similarly, for the probabilistic method cohsMix, we set the max iteration number to 200, chose the best result of cohsMix determined by maximum likelihood among 10 runs for each sample set, and then reported the average values and the standard deviations on 10 samples of each test setting.

In the group of experiments on real-world networks, we used the same parameter settings as in the original method publications in nearly all cases. We set *λ* = 5 for PCL-DC, PPL-DC, and PPSB-DC and *λ* = 1 for CESNA because these settings produced the best performance. We set the max iteration number to 10 for BAGC and GBAGC. We chose $\bar{K}=50$ for CODICIL because it resulted in the best performance among the options $\bar{K}\in \{30,50,70\}$. We set $\bar{D}$ of GLFM to 20. The weight between link structure and node attributes was 0.5 for cluster-dp and CODICIL. The max iteration number of cohsMix was 200 since we used only node attributes to make up for the sparsity of the original network and strengthen its community structure. The parameter *k* of a *k*NN attribute graph was also 10 for all real networks with the exception of the Diabetes data set, for which we set *k* = 60 due to the fact that there were only 3 large communities with thousands of nodes and a larger *k* provided better performance.
